# Robust Deep Neural Network for Learning in Noisy Multi-Label Food Images

**DOI:** 10.3390/s24072034

**Published:** 2024-03-22

**Authors:** Roberto Morales, Angela Martinez-Arroyo, Eduardo Aguilar

**Affiliations:** 1 Departamento de Ingeniería y Sistemas de Computación, Universidad Católica del Norte, Av. Angamos 0610, Antofagasta 1270709, Chile; roberto.morales02@alumnos.ucn.cl; 2 Centro de Investigación del Comportamiento Alimentario, Escuela Nutrición y Dietética, Universidad de Valparaíso, Av. Gran Bretaña. Playa Ancha, Valparaíso 2360102, Chile; angela.martinez@uv.cl; 3 Departament de Matemàtiques i Informàtica, Universitat de Barcelona, Gran Via de les Corts Catalanes 585, 08007 Barcelona, Spain

**Keywords:** learning with noisy labels, multi-label food recognition, MixUp, class activation map, bayesian statistics

## Abstract

Deep networks can facilitate the monitoring of a balanced diet to help prevent various health problems related to eating disorders. Large, diverse, and clean data are essential for learning these types of algorithms. Although data can be collected automatically, the data cleaning process is time-consuming. This study aims to provide the model with the ability to learn even when the data are not completely clean. For this purpose, we extend the Attentive Feature MixUp method to enable its learning on noisy multi-label food data. The extension was based on the hypothesis that during the MixUp phase, when a pair of images are mixed, the resulting soft labels should be different for each ingredient, being larger for ingredients that are mixed with the background because they are better distinguished than when they are mixed with other ingredients. Furthermore, to address data perturbation, the incorporation of the Laplace approximation as a post-hoc method was analyzed. The evaluation of the proposed method was performed on two food datasets, where a notable performance improvement was obtained in terms of Jaccard index and $F_{1}$ score, which validated the hypothesis raised. With the proposed MixUp, our method reduces the memorization of noisy multi-labels, thereby improving its performance.

## 1. Introduction

Obesity is a leading cause of non-communicable diseases (i.e., cardiovascular diseases, type 2 diabetes, and certain cancers.), and it has been estimated that globally, 11 million deaths and 255 million disability adjusted life years were attributed to behaviors and diets [1]. An accurate diet assessment is the key to understanding feeding at an individual level or population and its relationship to health. Nevertheless, dietary assessment is a challenge because the diet is subject to error via limitations of human memory, social desirability bias, and reactivity to self-monitoring (i.e., altered energy or food intake on reporting days, among other factors) [2]. The cross-disciplinary work of medical scientists, statistical engineers, and software developers from multiple domains has contributed to a deeper understanding of the relationship between diet and health. Developing some tools to assess or know feeding behaviors from individuals can enable self-monitoring and help health professionals indicate personalized professional recommendations [3].

In recent decades, advances in artificial intelligence have seen significant growth, both in the realms of research and in societal applications. This phenomenon has led to an increasingly substantial integration of this discipline into people’s daily lives. The food sector is no exception to this trend and has directly experienced the influence of artificial intelligence models, which have contributed to the improvement of metrics related to food recognition [4]. While these metrics are crucial indicators for evaluating model performance, it is essential to understand that they depend on various external factors, such as the size of the dataset, image quality, and model architecture, among others. A particularly relevant factor is the accuracy of label annotations. These annotations are carried out by external individuals and are based on their perception, which can lead to noisy or missing annotations. The annotation process, relying on individual interpretation, may introduce inaccuracies, and the class set may include snapshots that do not correspond to the designated category. This, in turn, negatively impacts the model’s performance, as it struggles to efficiently handle noise in datasets. Hence, it is crucial to develop models capable of effectively managing the noise present in annotations.

Deep networks have been successful in dealing with the noise present in the data to provide a robust solution for both non-sequential [5] and sequential data [6,7]. Specifically for the problem of learning with noisy labels, many of the deep learning-based studies have focused on the single-label paradigm [8,9]. These methods have not only demonstrated superior performance compared to their state-of-the-art (SoA) predecessors but have also served as catalysts for innovation, driving the incorporation of new processing techniques. Among these techniques are those utilizing probabilities [10] to enhance performance and those incorporating the MixUp technique [11,12], which blends labels to manage noise within them. Also, some approaches have addressed the management of noise in annotations through Bayesian and non-Bayesian approaches that introduce uncertainty into the training process [12,13,14,15]. This uncertainty is strategically employed to enhance the robustness and performance of models in challenging conditions, such as noise in labels and imbalanced distributions. Although there are several works proposed for single-label recognition, efficient handling of annotation noise has so far been recently explored in multi-label approaches [16,17,18], which have been validated on datasets with non-food images. In these works, novel techniques have been proposed to exclude noisy samples during backpropagation [16], reduce multi-label noise using their proposed StichUp augmentation [18], and learn label dependencies using graph convolutional networks [17].

In this work, the main difference lies in the multi-label perspective to address noisy annotations in food image recognition. This approach allows the model to recognize multiple foods simultaneously, which is crucial since most dishes tend to contain a variety of elements. By adopting a multi-label scheme, we provide the model with the ability to deal with this complexity more effectively. Additionally, by integrating noisy label management, we significantly improve the model’s performance against this common challenge in datasets. This drives us towards creating a robust model that can handle annotation noise, thereby strengthening its ability to perform precise and reliable recognitions in diverse food environments. Our proposed method, called attentive feature CAM-driven MixUp (AFCM), represents a significant evolution from previous approaches. AFCM uniquely addresses the blending between image regions where objects overlap and object-free areas. By utilizing activation maps generated by Grad-CAM++, AFCM extracts valuable information about the region containing the object to refine the attention weights used during label blending. This technique enables us to offer a significant improvement in the accuracy and adaptability of our models, providing more reliable and relevant results.

The main contributions are the following:

- We propose a novel method to deal with noisy multi-label learning for food recognition. The method is based on the AFM [19] and adapts the MixUp stage by considering the degree of overlap in the ingredients of a pair of images to generate the soft labels.
- We evaluate a probabilistic variant of the proposed method to analyze its robustness against data perturbations.
- We published a new dataset with foods belonging to the Chilean diet. The dataset contains multi-label annotations.
- We showed that the proposed method was less affected by noisy labels and outperformed the rest of the methods on two food datasets.

The remainder of this article is organized as follows: Section 2 presents the literature related to the research developed. Section 4 presents a description of the proposed method. Section 5 details the experimental setup, including the datasets used, the model implementation, and the evaluation metrics. Section 6 shows the results. Section 7 and Section 8 are the discussion and conclusions, respectively.

## 2. Related Work

In this section, we conduct a comprehensive analysis of the literature related to the research we have developed. We focus on the progress made in the field of deep learning models applied to food recognition, as well as the identification of multiple objects considering the presence of noisy labels. We explore the most relevant studies that have contributed to the development of these models, highlighting their approaches, methodologies, and results. This review provides the necessary context to understand the unique and original contribution of our work in this constantly evolving area.

### 2.1. Noisy Single-Label Visual Food Recognition

In the realm of deep learning, one of the most common challenges faced is the presence of noise in training labels, which affects the quality and accuracy of image classification models. To tackle this issue, various approaches and methods have been devised and evaluated on image datasets, particularly focusing on single-label image classification.

Several methods have been developed that address different perspectives. One example is CleanNet [8], which introduces an innovative approach to mitigate label noise during the training of large-scale image classification models. By identifying label noise and examining only a fraction of classes, CleanNet efficiently transfers this information to other classes, thus minimizing the need for human intervention. Co-learning [9], on the other hand, stands out as a method that merges supervised and self-supervised learning by using a shared feature encoder and implementing similarity constraints to enhance performance in contexts with noisy labels. Experiments were conducted with CIFAR-10 [20], CIFAR-100 [20], Animal-10N [21], and Food-101N [8]. Significant improvements in accuracy were observed in this process, both with symmetric and asymmetric noise, resulting in noteworthy accuracies of 92.21% for CIFAR-10, 91.07% for CIFAR-100, 66.58% for Animal-10N, and 65.26% for Food-101N. Another method that combines supervised and self-supervised learning is LongReMix [11], which is based on an unsupervised learning stage to classify clean and noisy training samples, followed by a semi-supervised learning stage to minimize the error variability rate (EVR) using a labeled set formed by samples classified as clean and an unlabeled set with samples classified as noisy. This enhanced the generalization ability of deep neural networks achieving a performance of 82.52% and 72.50% in Food-101N and Clothing1M, respectively.

Researchers have also focused their attention on meta-learning, where MSLG [22] introduces a meta-learning approach that seeks the optimal distribution of soft labels aligned with a meta-objective: minimizing loss on the small meta dataset. The network undergoes training using these predicted soft labels. This two-stage process is repeated throughout the training process. The model stands out for its independence from the type of backbone used, achieving an accuracy of about 75% in CIFAR-10, even in extreme conditions with 80% feature-dependent noise. It also requires few metadata; only 1000 metadata achieve the best performance on CIFAR-10 with 50,000 noisy data. In the case of Food101-N, MSLG surpasses several SoA methods. Continuing in the meta-learning line, WarPi [23] proposes a model designed to adaptively learn the correction of the training process in a meta-learning context. It also incorporates a probabilistic approach by conceiving the learning process as a hierarchical probabilistic model. Considering the correction vector as a latent variable allows for effective estimation of the predictive posterior. The consistently obtained results outperform other meta-learning methods such as L2RW [24], MWNet [25], and MLC [26]. In the scenario of CIFAR-100 with 40% asymmetric noise, WarPi surpasses MWNet by 3.73%.

On the other hand, CoDis [27] uses the discrepancy between two neural networks, employing Jensen-Shannon (JS) divergence, to select examples likely to be clean. CoDis overcomes sample selection inefficiency, improves generalization, and excels in the robustness of models to noisy labels, especially on imbalanced datasets. This approach is not only efficient in terms of samples but also excels in extracting hard samples, which are crucial for generalization. Experimental results reveal the effectiveness of CoDis on noisy datasets, whether balanced or imbalanced. JS divergence was also adopted in Jo-SRC [28] to measure the likelihood that a sample is clean. Additionally, JS was applied to distinguish if noisy samples belonged to the in-distribution (ID) or out-of-distribution (OOD). It introduces a joint loss that combines classification and consistency regularization terms, resulting in substantial improvements in model performance and robustness against noisy labels. In Food-101N, Jo-SRC stands as a leader with an impressive 86.05%. The categorization of the noise in ID and OOD was also made in PNP [10]. In this case, based on a probabilistic approach, PNP employs two networks to anticipate both the category and type of noise. It introduces a regression task to improve accuracy in predicting the type of noise and applies consistency regularization to enhance discrimination capacity. Experimental results, conducted on both synthetic and real-world datasets, highlight the remarkable effectiveness of this method. Both PNP-Hard and PNP-Soft achieved test accuracy of 87.31% and 87.50%, respectively, on the Food-101N dataset. These results outperform leading approaches, demonstrating the method’s effectiveness in large-scale real-world applications.

From a different perspective, the attentive feature MixUp (AFM) method [19] tackles the challenge of noisy labels in deep learning models by assigning attention weights to samples. This strategy allows models to focus more on clean samples and reduce attention to noisy samples. AFM improves sample quality by interpolating samples grouped with attenuated noise. Unlike other approaches [8,11], it does not require additional clean datasets and jointly optimizes interpolation weights along with classifiers. Experiments highlight the outstanding performance of AFM on real-world datasets with noisy labels, such as Food-101N and Clothing1M. Another efficient approach, recently published, is known as Dynamic Instance Selection and Correction (DISC) [29]. DISC employs augmentation techniques to generate two equivalent views and use them to select reliable instances and correct noisy labels. The method optimizes learning on noisy datasets through a dynamic instance-specific threshold strategy to divide noisy data into three subsets: clean, noisy, and purified. Then, different regularization techniques are adapted to compute the loss. Conventional cross entropy for the clean set, generalized cross entropy [30] to handle noise labels, and binary cross entropy for the formed set by the union of the three subsets using the MixUp approach. Experiments demonstrate that DISC outperforms multiple baselines in noisy and balanced datasets, showcasing competitiveness and efficiency in sample usage.

### 2.2. Uncertainty-Aware Noisy Single-Label Visual Recognition

Lately, some approaches have emerged that incorporate uncertainty, improving the robustness of learning with noisy labels and therefore their results. The few methods found can be grouped into two: non-Bayesian methods [13,14], which use prior networks [31] or mixtures of experts, and Bayesian methods [12,15,32], which are based on the MC-Dropout approximation [33].

In [13], the unsupervised confidence approximation (UCA) framework was proposed, which enables the concurrent training of the main task and confidence prediction without confidence labels. UCA weighs the contribution of each sample to the loss based on prior weight distribution, down-weighting those prone to noise. Here the confidence predicted is treated as a measure of prediction uncertainty. Another non-Bayesian method, called mixture logit networks (MLN), [14], is proposed to address the challenge of learning from noisy and corrupt training data by identifying specific corruption patterns. MLN not only successfully learns the clean target distribution from dirty data but also estimates the underlying pattern of noise. It utilizes a mixture of expert models to distinguish between aleatoric and epistemic uncertainty, achieving robustness and explainability in classification. The method excels in high-corruption environments (e.g., 80% of noise), outperforming others on datasets such as MNIST, CIFAR10, and CIFAR100. Experiments highlight the influence of uncertainty regularizers on MLN performance, emphasizing its unique efficacy in various noise settings.

On the other hand, a novel approach called dynamic loss [15] was proposed to jointly address the problems of data imbalances and noisy labels. This loss adapts during training, comprising a label corrector and a margin generator. The former corrects noisy labels, while the latter produces class-wise classification margins, capturing the underlying distribution of data and the state of the classifier. Extensive evaluation of synthetic and real-world datasets demonstrates leading performance across various assessments. A Bayesian variant, by incorporating MC-Dropout, of dynamic loss was also evaluated, achieving the best accuracy on CIFAR-10N. These results suggest that uncertainty can serve as a significant complement to its proposal for learning with noisy labels. Challenges in memorization and label uncertainty were addressed for the learning algorithms in the presence of noisy labels by the method named Bayesian DivideMix++ [12]. The proposed solution consists of two key components: DivideMix++ to mitigate memorization and Monte-Carlo MixMatch to enhance effectiveness in the face of label uncertainty. Key contributions focus on substantial improvements to the DivideMix algorithm, emphasizing analysis in pre-training, augmentation strategies, and handling uncertainty in annotations during the MixUp phase. Evaluation of the proposal is conducted on diverse benchmarks and real-world datasets, revealing improvements compared to SoA methods. Finally, a robust and efficient method called single dropout for noisy labels (USDNL) was proposed in [32]. USDNL uses the MC-Dropout approach to estimate the epistemic uncertainty of the network’s predictions, combining it with prediction cross-entropy to select clean samples during training. Unlike previous approaches, USDNL does it with a single dropout, significantly reducing computational complexity. The method was proven to be effective and efficient in mitigating noisy labels, both in artificial noise and real-world environments.

### 2.3. Noisy Multi-Label Visual Recognition

Few deep-learning methods have been proposed to address multi-label object recognition with noisy annotations. These methods are based on a co-learning approach [16,18] or provide novel loss formulation by modeling the complex correlations between labels [17,34,35]. In all cases, the performance is improved compared to methods that do not consciously take into account the noise in the labels of the dataset.

As for co-learning, a framework for addressing long-tailed multi-label visual classification with noisy labels was proposed in [18]. As part of their framework, they propose the effective Stitch-Up technique, used to synthesize cleaner training samples by concatenating images within the same classes, reducing noise from a probabilistic justification. In addition, the proposed co-learning framework considers training two branches jointly to correct noisy labels, taking into account two sampling strategies (random and balancing) to provide high performance on both head and tail samples. On the other hand, in [16], the RCML method was proposed as a pioneering solution to automatically handle two types of noise in multi-label images without prior assumptions, which are missing labels and wrong labels. The method is based on three modules: (1) the discrepancy module, which ensures the two networks learn diverse features; (2) the group lasso module to detect potential noisy labels; and (3) the swap module to exchange the ranking information between networks. Interesting findings extracted from the results reveal that the error in the wrong label is more detrimental than the absence of a label.

The importance of analyzing label dependency to distinguish examples with clean/noise multi-labeling was raised in [17]. For that purpose, the author provides an algorithm called holistic correction for multi-label classification with noisy labels (HLC). This algorithm leverages the memorization effect to learn the dependence between labels, exclusively using training data containing multiple noisy labels. The holistic score is introduced in HLC to measure instance-label and label dependencies in an example. Based on this score, likely noisy labels can be corrected during the holistic correction phase. In [34], they propose the method called trusted loss correction for noisy multi-label learning (TLCM), which estimates the label corruption matrix in multi-label classification by taking into account label dependency and imbalance. TLCM uses a gold loss correction by leveraging a small set of reliable data to estimate the noise corruption matrix, which is then used to correct the model loss during training. This method incorporates individual label regulators to improve the accuracy of the multi-label classifier, thus overcoming the challenges associated with label noise. Another approach formalizes the class-conditional multi-label noise (CCMN) [35]. They propose a modified loss of the ranking loss, taking into account the assumption that there are correlations between labels in a multi-label problem. A special case of the CCMN framework is the proposed new approach called the unbiased partial multi-label learning (uPML) estimator, which proves to be effective on multiple datasets.

From the literature review, no previous methods for dealing with multi-label learning with noise in food images are apparent. In other domains, co-learning and loss-based label dependency learning methods have been effective. Although MixUp has been successfully used in noisy single-label learning, it has not yet been evaluated for multi-label learning. Considering its cost-effectiveness, its flexibility to adapt to the multi-label problem, and the good results obtained on food images, we selected the MixUp-based method called AFM and extended it appropriately for the noisy multi-label learning problem. Moreover, motivated by uncertainty-aware approaches, we will explore their effectiveness in improving the robustness of the problem at hand.

## 3. Background

This section provides a concise overview of the AFM and Grad-CAM++ methods, providing background information to understand the main components of the proposed method.

### 3.1. Attentive Feature MixUp for Learning with Noisy Labels

The AFM has several appealing benefits for robust deep learning in the presence of noise. Firstly, it does not rely on assumptions or an additional clean subset. Secondly, with massive interpolations, the ratio of useless samples is drastically reduced compared to the original noisy ratio. Thirdly, it jointly optimizes interpolation weights with classifiers, suppressing the influence of mislabeled data through low attention weights. Fourthly, it partially inherits the vicinal risk minimization of MixUp to alleviate overfitting, improving it by sampling fewer feature-target vectors around mislabeled data from the vicinal distribution of MixUp. The AFM method focuses on two main modules: group-to-attend (GA) and MixUp. GA significantly reduces the impact of samples with noisy labels and decreases the proportion of completely noisy groups. In addition, it allows partially noisy groups to provide useful monitoring through the well-trained attention network. Since a large number of group members may result in excessively smoothed features, which is detrimental to discriminative feature learning, in our proposal, 2-member groups ($x_{i}$ and $x_{j})$ were used. Regarding the MixUp [36], this module interpolates virtual feature-target vectors during training. Adhering to the traditional MixUp approach, the attention weight ($a_{i}$) is scaled through a sigmoid function to fall within the range [0, 1]. Therefore, formally, MixUp can be expressed as follows:

(1) $$x=a_{i}\cdot x_{i}+\left(1-a_{i}\right)\cdot x_{j}$$

(2) $$y=a_{i}\cdot y_{i}+\left(1-a_{i}\right)\cdot y_{j}$$

where [$x_{i}$, $x_{j}$] and [$y_{i}$, $y_{j}$] are the interpolated features and soft labels, respectively, for the group consisting of the *i*-th and *j*-th data.

The total loss ($L_{total}$) is composed of two parts: $L_{org}$, which considers the logit output of a fully connected (FC) layer receiving as input the original features of the last convolutional layer; and $L_{afm}$, which considers the logit output of another FC layer receiving the features of the interpolated data. The loss can be expressed as follows:

(3) $$L_{total}=\lambda \cdot L_{afm}+\left(1-\lambda \right)\cdot L_{org}$$

AFM was designed for single-label classification, and for this reason, categorical cross-entropy with softmax activation was used in both losses ($L_{afm}$ and $L_{org}$). After analyzing the components, like any benchmark deep network, we observed that AFM can be easily adapted to multi-label classification by adapting the loss function to binary cross-entropy and using sigmoid activation on the logit outputs.

### 3.2. Grad-CAM++ for Unsupervised Object Localization

Grad-CAM++ [37] is an extension or enhancement of Grad-CAM [38], which is used as a visualization technique to understand which specific regions of an image contribute more to the final prediction performed by a convolutional neural network (CNN) model. The main improvement of Grad-CAM++ over Grad-CAM is that it considers not only positive gradients but also negative gradients when calculating the importance of each pixel. This addition is intended to provide a more accurate and detailed visualization of regions of interest in the image, as it captures both the significance of positive evidence for a given class and the lack of significance for other classes. Unlike Grad-CAM, Grad-CAM++ can be used in post-processing after the neural network forward step. The gradients of the last convolutional layer are combined to generate the class activation map. Given an input image and a target class, Grad-CAM++ highlights in the activation map the regions that the model considers most important for making the decision.

**Figure 1:**
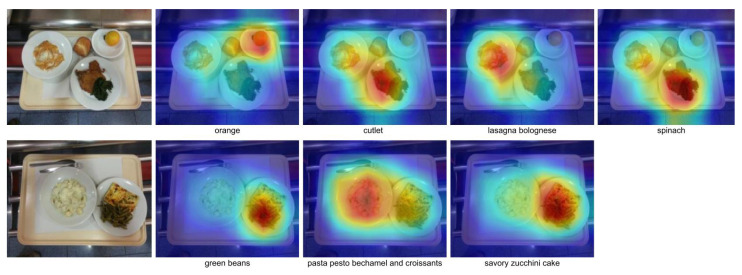
Example Grad-CAM++ using AFCM. This example demonstrates the utilization of Grad-CAM++ in AFCM, showcasing the capability of the model to discern the regions of the targeted food.

A sample of the maps generated by Grad-CAM++, using the proposed method, for each ground truth label belonging to the input image can be seen in Figure 1. For this sample, Grad-CAM++ effectively identifies the specific areas where food is found. These results demonstrate the ability of Grad-CAM++ to determine food areas in a semi-controlled environment, so it can be considered a good alternative for unsupervised object localization.

## 4. Attentive Feature CAM-Driven MixUp

In this section, we explain the proposed method for learning with noisy multi-label data, detailing how AFM and Grad-CAM++ are integrated into the proposed approach. In addition, we introduce the Laplace approximation that allows the model to learn the posterior distribution. The latter can be used at the time of inference as a post-hoc method to improve the model’s robustness when the data differ from the learned distribution.

### 4.1. Rationale

The proposed method attentive feature CAM-driven MixUp (AFCM) is an extension of the AFM method to handle multi-label data. AFM was selected due to the good performance demonstrated on food datasets for the noisy label learning problem. AFM has been designed for single-label problems, that is, when images are labeled by the general content. In this context, noisy labels may be due to a weakly supervised data acquisition process or to errors by the labelers. A straightforward way to use it to perform multi-label classification is by modifying the loss and activation functions of the output layer. Specifically, replacing softmax activation with sigmoid activation to ensure independent probabilities and optimizing binary cross-entropy loss instead of categorical cross-entropy loss. However, noisy labels may present differently on single labels compared to multi-label problems. Unlike the single-label, where the noise is related to the general content, in the multi-label, the noise may appear due to missed labels or wrong labels in some or all of the annotations assigned to the image. Therefore, noisy multi-label learning should not be treated in the same way.

AFM mitigates the error in the learning process produced by noisy labeled data by generating softly labeled virtual features based on the MixUp module. During the MixUp phase, the features extracted from a group of images and the corresponding labels are mixed in different proportions using attention weights. We argue that for multi-label problems, we should not use a single attention weight for all labels, so we decided to redefine the MixUp module to work correctly for the task at hand. The reason is that when two images are mixed, not always all ingredients are mixed; in some cases, the area of overlap may be between the ingredient of one image and the background of the other. We hypothesize that ingredients that combine with the background might be easier to learn and recognize than those that combine with other ingredients.

**Figure 2:**
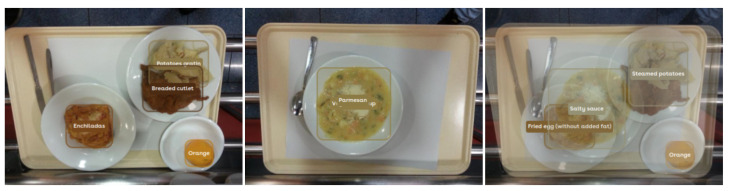
Recognition results of mixed dishes placed on two individual food trays (**left** and **center**) and a synthetic image generated by blending these trays (**right**).

To illustrate how the performance of the model is affected when two food images are blended, we performed the analysis of three images: two images of real food trays and a third one corresponding to a blend of the real ones. Figure 2 shows the results obtained in food recognition by the Foodvisor platform, which is the one that provides the best results in the recognition of mixed dishes [39]. The food recognition is not quite correct; however, the interesting thing about this is to observe how it affects performance when two foods are blended or when a food is blended with an object-free area. The results provided by the platform reaffirm our hypothesis that both foods blended with the background, the potato-based side dish and the orange, maintain a correct prediction as opposed to the blended foods, which are completely wrong.

Based on the stated hypothesis, the location of the ingredients in food images should be determined and used during soft label generation to provide individual labels for each ingredient. However, in the multi-label problem, the annotations are related to the ingredients contained in the image, but there is no information about the locations. In our proposal, to estimate the ingredient locations, we decided to use an explainability algorithm like Grad-CAM++ because, as was observed in [39,40], Grad-CAM++ is a good alternative to estimate the object location and segmentation in an unsupervised way and can be used at the top of a neural network without retraining.

### 4.2. Rethinking MixUp in the AFM Method for Learning from Noisy Multi-Labels

**Figure 3:**
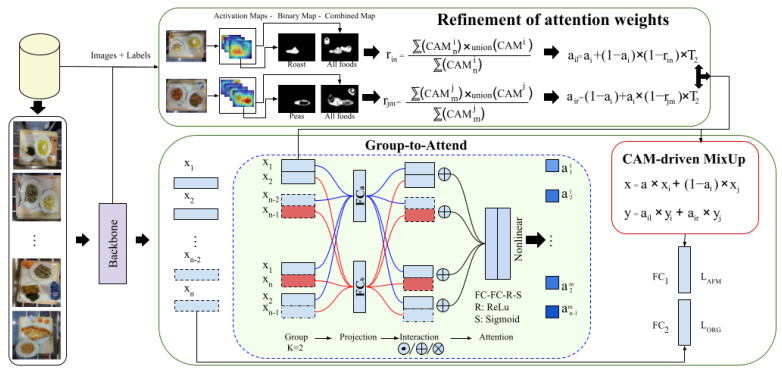
The pipeline of attentive feature CAM-driven MixUp (AFCM) consists of several fundamental steps. First, a CNN backbone is applied to a mini-batch of images to extract relevant features. Subsequently, a group-to-attend (GA) module forms groups of a predefined size K=2 randomly. Within each group, every element undergoes linear projection using a dedicated fully connected (FC) layer. The elements of each group are then combined, generating a set of K attention weights for each group. These weights, representing the relative importance of each group, are utilized in a CAM-driven MixUp module. This is where interpolation takes place to create new samples and soft labels, leveraging the information provided by class activation maps (CAM).

This subsection details how MixUp is redefined in AFM based on the proposed approach to generate soft labels for multi-label problems. As can be seen in the in Figure 3, both AFM and Grad-CAM++ form the basis of the proposed AFCM method. AFCM evolves from the previously developed AFM method by considering differently the blending between image regions where objects overlap and when the overlapping involves an object-free area (background). With the help of Grad-CAM++, valuable information about the region containing the object was extracted to refine the attention weights used during label blending. Note that, Grad-CAM++ can be interchanged with another method that allows us to detect food areas without supervision.

During training, after each forward step, the trained model, input images, and corresponding labels are passed to the proposed Refinement of the Attention Weights module to generate the specific weight for each ingredient, subsequently used in soft label generation (see the upper part of Figure 3). Let’s consider the activation maps $CAM^{i}=${$CAM_{1}^{i}$, …, $CAM_{N}^{i}$} and $CAM^{j}=${$CAM_{1}^{j}$, …, $CAM_{M}^{j}$} generated by Grad-CAM++ for i-th and j-th images with *N* and *M* targets, respectively; and then binarizing them, leaving the areas where objects are likely to be present, by normalizing the gradient and applying an indicator function that results in 1 when the values are greater than $T_{1}=0.5$ and 0 otherwise. The attention weight $a_{i}$ is redefined as follows:

(4) $$a_{il}=a_{i}+\left(1-a_{i}\right)\cdot \left(1-r_{in}\right)\cdot T_{2}$$

(5) $$a_{ir}=\left(1-a_{i}\right)+a_{i}\cdot \left(1-r_{jm}\right)\cdot T_{2}$$

where $a_{il}$ and $a_{ir}$ represent the vectors with the weights for the targets $y_{i}$ and $y_{j}$, respectively; $T_{2}$ a threshold to regularize how much the weight increases, and *r* the overlap ratio for the n-th and m-th target belonging to the i-th and j-th image calculated by the following formula:

(6) $$r_{in}=\frac{\sum \left(CAM_{n}^{i}\cdot \mathrm{union}\left(CAM^{j}\right)\right)}{\sum CAM_{n}^{i}}$$

(7) $$r_{jm}=\frac{\sum \left(CAM_{m}^{j}\cdot \mathrm{union}\left(CAM^{i}\right)\right)}{\sum CAM_{m}^{j}}$$

where union(.) is a function that performs an element-wise comparison between arrays and returns an array with 1’s in each position where at least one of the arrays has 1 in this position and 0 otherwise.

Finally, the soft labels are calculated as follows:

(8) $$y=a_{il}\cdot y_{i}+a_{ir}\cdot y_{j}$$

Note that when $T_{2}$ takes a value equal to 0, the proposed AFCM method becomes the original AFM. On the other hand, when $T_{2}$ takes a value equal to 1, the attention weight ($a_{i}$) for a particular ingredient can be equal to 1 if the ingredient in one image completely overlaps the background of the other image, equal to $a_{i}$ when completely overlapping a food area and between $a_{i}$ and 1 depending on the overlap ratio.

### 4.3. Probabilistic AFCM for Robust Prediction

In addition to extending AFM to adequately address noisy multi-label learning, motivated by the literature review [12,13,14,15], we explore incorporating an additional component into the proposed AFCM to provide robust prediction on data with inherent noise that may occur during the data acquisition process. The literature review described Bayesian and non-Bayesian approaches capable of quantifying uncertainty and thus improving robustness to deal with noisy data or labels. Non-Bayesian approaches have been discarded because they are more difficult to model than the Bayesian approaches reviewed. Therefore, a probabilistic variant of the AFCM, based on Bayesian statistics, is proposed to deal with complex data in noisy multi-label learning problems. Inspired by [41], where the Laplace approximation (LA) [42] was effectively applied to improve the results in multi-label classification on a clean dataset, we propose to integrate it into the AFCM method. Unlike other probabilistic methods such as MC-Dropout [33] and Bayes by Backprop [43], the LA can be used as a post-hoc method on an already-trained neural network [44] to approximate the posterior probability distribution.

Let θ be the weights of the neural networks and D be the data seen. In LA, the posterior distribution is approximated as Gaussian:

(9) $$p\left(\theta |D\right)\approx N\left(\theta ;\theta_{MAP},\;H^{-1}\right)$$

Since the mean, $\theta_{MAP}$, corresponding to the maximum a posteriori, is obtained by the traditional deep learning training procedure; the only additional step corresponds to the computation of the covariance, $H^{-1}$, which corresponds to the inverse of the Hessian matrix of the negative log posterior [41,44].

Once $H^{-1}$ is calculated, the posterior predictive distribution $p(D^{*}|D)$) on unseen data ($D^{*}$) can be approximated with Monte Carlo integration using *K* samples $\theta_{k}$ from the approximated posterior as follows [42]:

(10) $$p\left(D^{*}|D\right)=\int p\left(D^{*}|\theta \right)p\left(\theta |D\right)d\theta \approx \frac{1}{K}\sum_{k=1}^{K}p(D^{*}|\theta_{k})$$

Daxberger et al. [44] detail 4 components that must be defined to incorporate LA in deep neural networks: (1) Weights to be treated probabilistically with Laplace, (2) Hessian approximation, (3) Hyperparameter tuning method, and (4) method to calculate the posterior predictive distribution. By considering the cost-effectiveness and following the LA integration for multi-label proposed in [41], we decided to treat only the weights of the last layer of AFCM as probabilistic; the Hessian approximation is made by Kronecker Factored Approximate Curvature (KFAC); the hyperparameter tunning is done by marginal likelihood maximization; and the posterior predictive is approximated by the Monte Carlo method.

## 5. Validation

This section describes the datasets and experimental setup used in the experimentation.

### 5.1. Datasets

To validate the proposed method, two datasets with multi-food images were selected, one acquired under semi-controlled conditions (UNIMIB2016) and the other with images collected from the web (MLChileanFood). UNIMIB2016 provides an ideal scenario to evaluate our hypothesis, in which different ingredients from one image can blend with other ingredients or with the (nearly smooth) background of the other image. MLChileanFood proves more challenging than UNIMIB2016, which is useful to determine how well our approach works on images acquired in the wild. Both datasets, UNIMIB2016 with noisy labels (UNIMIB2016-N) and MLChileanFood, are described by providing complete information on the nature, origin, and specific characteristics of each dataset to provide a complete context for the development and interpretation of our experiments.

#### 5.1.1. UNIMIB2016-N

This dataset arose in response to the need to evaluate how models handle noise in annotations, based on information gathered under semi-controlled conditions in UNIMIB2016. We focus specifically on the foods present in each tray (but not their location), paying particular attention to the 73 Italian food categories contained in this dataset. The original annotations were altered by randomly substituting 20% of the foods in the training set, resulting in a total of 132 foods with noisy annotations. This procedure transformed UNIMIB2016 into a dataset with 20% noise in its annotations, leading to the creation of UNIMIB2016-N. This initiative allows for a more rigorous assessment of the model’s ability to deal with the variability and inaccuracies inherent in the food annotation process. UNIMIB2016-N is composed of a total of 1010 images of trays with multiple foods, of which 650 are for training and the remaining 360 for testing. We maintain the same division of the datasets as the UNIMIB2016 datasets.

#### 5.1.2. MLChileanFood

The main purpose behind creating this dataset was to capture images of combo plates in uncontrolled conditions. Specifically, this dataset is focused on the dietary preferences of university students in Chile. To achieve this, two teams were formed: one at the Catholic University of the North and another at the University of Valparaiso. These teams dedicated themselves to photographing and thoroughly describing their main meals over a month. Subsequently, they delved into each documented dish, compiling a detailed list of combo plates, resulting in a total of 155 combinations and 183 ingredients identified. Using advanced web scraping techniques, images were collected using the names of the combo plates as query terms, about 73 images per query, yielding a dataset comprising 12,300 images. Finally, a filter was applied to the ingredients to select only those that were represented in more than 10 instances in the dataset, resulting in a total of 114 ingredients among all images. For training purposes, the dataset was divided into 80% of the images for training and the remaining 20% for testing. Considering that the images are grouped by the query term used, the division was performed randomly over each group to provide a similar distribution of ingredients in both sets.

Note that the annotation phase was conducted by an external labeler with experience in multi-label annotations. Each food image was individually annotated by this labeler. However, considering that both the image collection and annotation processes inherently involve inevitable errors, a new dataset is likely to have noise in the annotations.

### 5.2. Implementation Details

For comparative purposes, all experiments have the same setup. Three deep neural networks are evaluated: ResNet-50, AFM, and the proposed AFCM. Both AFM and AFCM use ResNet-50 as a backbone. Before training began, ResNet-50 was initialized with the ImageNet weights. The neural networks were then trained for 100 epochs using a batch size of 32. The optimization was performed employing the stochastic gradient descent (SGD), with an initial learning rate (LR) of 0.01 and momentum of 0.9. Regarding the regularization, a weight decay of $1\times 10^{-4}$ was used. The LR decreases following a multi-step policy; specifically, the LR is multiplied by 0.1 at epochs 50 and 75.

As for data processing, images were resized to 224 and normalized by subtracting the mean ([0.485, 0.456, 0.406]) and dividing by the standard deviation ([0.229, 0.224, 0.225]). Regarding data augmentation (DA), during training, only a random horizontal flip was used.

On the one hand, for the probabilistic variant of AFCM, a Monte Carlo sampling of 10 forward passes was applied to estimate the mean prediction. On the other hand, to weigh the importance of both components in the loss function, we use a λ value equal to 0.75.

For the evaluation, the methods were trained five times, and the mean and standard deviation were reported using the traditional metrics for multi-label classification. Specifically, precision (P), recall (R), $F_{1}$ score, and Jaccard index (JI). Each of these metrics provides a specific evaluation of the model’s performance: P measures the ability of the model to generate fewer false predictions; R measures the ability of the model to detect all labels present in the image; $F_{1}$ provides a balance between P and R; and JI measures how well the model predictions fit the correct labels using the intersection over the union of them.

Finally, all experiments were conducted on a server with a graphics card (NVIDIA GeForce RTX 4090 (Santa Clara, CA, USA)) using the Python framework PyTorch 2.0.1 as the main platform, leveraging its versatility and power in deep learning.

## 6. Results

This section presents the experimental results used to evaluate the performance of the proposed AFCM method.

### 6.1. AFCM Threshold Analysis

**Figure 4:**
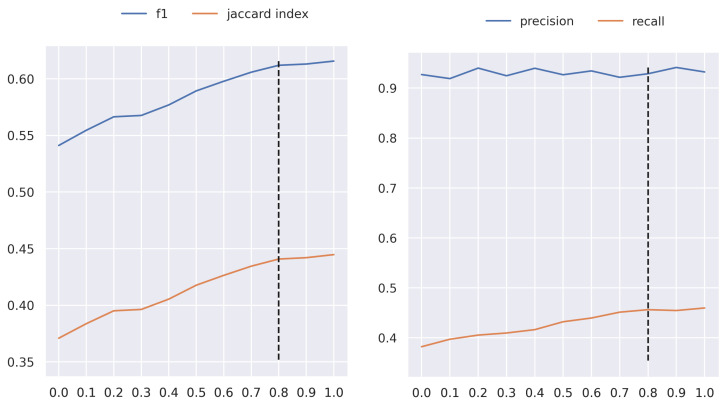
Performance of the AFCM method in UNIMIB2016-N, in terms of $F_{1}$ and JI, varying the threshold from 0 to 1 in increments of 0.1. The black dotted line indicates the selected threshold.

The proposed AFCM considers that some ingredients must be weighted more than others during the MixUp phase. This depends on the overlap degree between other ingredients or backgrounds. The weight obtained by the neural network is redefined for each ingredient and increases as the overlap is greater in the background area. The increase depends on the threshold used, which can vary from 0 to 1. Figure 4 presents the results in terms of $F_{1}$ score and Jaccard index in UNIMIB2016-N, when the threshold takes a value from 0 to 1 with a step of 0.1. As can be seen, the performance tends to increase as the threshold is raised. This behavior is maintained until the value of 0.8, where the result stabilizes. That is the reason that we use this value for the rest of the experiments.

### 6.2. Comparative Analysis of Method Performance

The AFCM method is validated on two food datasets, UNIMIB2016-N and MLChileanFood, and compared with ResNet-50 (considered as baseline) and the AFM corresponding to the method used as the basis of our proposal. The results in terms of recall (R), precision (P), $F_{1}$ score, and Jaccard index (JI) can be seen in Table 1 in UNIMIB2016-N. From this, it is observed that using AFM * for the multi-label problem, i.e., changing the loss function to binary cross-entropy and activation to sigmoid, immediately shows an improvement in all metrics of approximately 3%, except for P, which remains almost the same. This reinforces our assertion that the components involved in AFM are also useful for noisy multi-label learning. The improvement is even more remarkable with the proposed AFCM, where it outperforms the other methods by far. Specifically, the increase is more than 10% in all metrics, except for P, where performance drops by around 2%. Therefore, knowing whether the ingredients are mixed with other ingredients or with the background positively influences the model’s performance.

**Figure 5:**
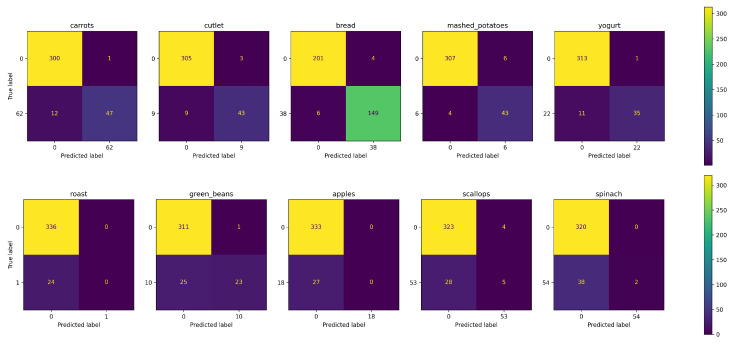
Confusion matrix for AFCM method performance in UNIMIB2016-N for the five labels with the most true positives (**top**) and false negatives (**bottom**). The numbers on the axes correspond to the class identification, where 0 means all other classes.

**Figure 6:**
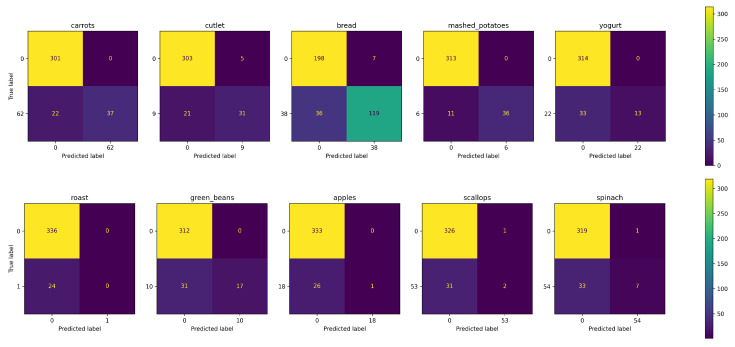
Confusion matrix for ResNet-50 method performance in UNIMIB2016-N for the five labels selected according to the most true positives (**top**) and false negatives (**bottom**) obtained by AFCM. The numbers on the axes correspond to the class identification, where 0 means all other classes.

To further analyze the model results, five confusion matrices were generated for the ingredients with the most true positives (TPs) and false negatives (FNs), selected according to the AFCM results. TPs and FNs can be observed in the first and second row, respectively, of Figure 5 for AFCM and Figure 6 for ResNet-50. In each confusion matrix, the upper left cell corresponds to true negative (TN), the upper right cell to false positive (FP), the lower left cell to FN, and the lower right cell to TP. In most cases, the FP is observed to be relatively small, which is consistent with the high P achieved by both models. In terms of TP, there is a great improvement in the ingredients in which AFCM provides its best performance. Most of these ingredients are side dishes, desserts, and bread, which have the most instances in the dataset. In terms of FNs, AFCM tends to have fewer than ResNet-50. All these aspects highlight the advantages of the proposed approach to learning with multi-label noise.

In addition to UNIMIB2016-N, the models were evaluated at MLChileanFood. Unlike UNIMIB2016-N, where noise was introduced synthetically to the labels, in MLChileanFood, it is assumed that noise can occur by the traditional labeling procedure. Furthermore, MLChileanFood is more challenging because the data are acquired from the Google search engine and therefore under unrestricted conditions. Table 2 shows the mean and standard deviation of the results obtained by the methods. Again, an improvement is observed with AFM * compared to ResNet-50 in all metrics except for P, where the performance drops about 3%. This suggests that the AFM can find more ingredients present in the image than ResNet-50, but in some cases, it predicts a few more ingredients that do not appear there. With AFCM, even better performance was observed for multi-label classification by providing a better balance between R and P and also a higher JI. The latter means that the models tend to produce fewer false predictions and more true predictions compared to the other methods.

The proposed AFCM was also compared with SoA methods developed to deal with noisy multi-label learning and validated on non-food image datasets. HLC [17] and CLMSU [18] were trained on UNIMIB2016-N and MLChileanFood using the same experimental setting proposed by the authors. The only change made was a reduction of the batch size used in HLC to 32 images to perform the training on the UNIMIB2016-N dataset, and a different number of samples was considered to generate the head, middle, and tail subsets in CLMSU, depending on the target dataset. Specifically, in UNIMIB2016-N, classes with more than 75 samples, less than 15 samples, and between 15–75 samples were considered to form the head, tail, and middle subsets. In MLChileanFood, more than 1000, less than 100, and between 100–1000 were considered to form the head, tail, and middle subsets. All methods (HLC, CLMSU, and AFCM) use the same backbone (ResNet-50) but differ in the optimizer or the training procedure. The results in terms of R, P, $F_{1}$, and JI are reported in Table 3. Although HLC and CLMSU provide comparable results on the MS-COCO object recognition benchmark dataset, the performance obtained on images belonging to the food domain differs over a wide range. CLMSU stands out for its ability to adapt to data from different domains. Overall, our AFCM method outperforms CLMSU in UNIMIB2016-N and provides comparable (slightly better) results in MLChileanFood. Focusing on P and R, it can be seen that AFCM provides about 9% less recall but more than 15% improvement in P for both datasets. These results show that our method provides a better trade-off in the reduction of false positive and false negative predictions, prioritizing the former. In summary, the proposed AFCM was able to provide better performance compared to the baseline (ResNet-50), the basis model (AFM), and the two SoA object recognition models.

In addition to the standard multi-label recognition metrics, a statistical significance analysis was performed using the Bonferroni-Dunns [45] test. The objective of this test is to determine whether the performance of the proposed AFCM method is significantly different from the other methods evaluated. Table 4 shows the test results when evaluating two classifiers, the proposed AFCM compared to ResNet-50, AFM, HLC, and CLMSU. To perform the test, sample-level food recognition results were obtained in terms of $F_{1}$ for UNIMIB2016-N and MLChileanFood. In most cases, the results provided by AFCM are significantly different. Interestingly, it is noted that, for MLChileanFood, although AFCM has almost the same performance difference as AFM and CLMSU (see Table 2 and Table 3), statistical significance is observed in CLMSU but not in AFM. This is in agreement with the precision and recall obtained by these methods. Specifically, CLMSU shows a completely different behavior, providing similar results in both metrics, unlike AFM and AFCM, which present a wide distance between them. Therefore, although CLMU has a slightly worse overall $F_{1}$ performance, at the sample level, the results are very different. In the case of the AFM, the increase in precision achieved by the AFCM was not sufficient to pass the statistical test.

**Figure 7:**
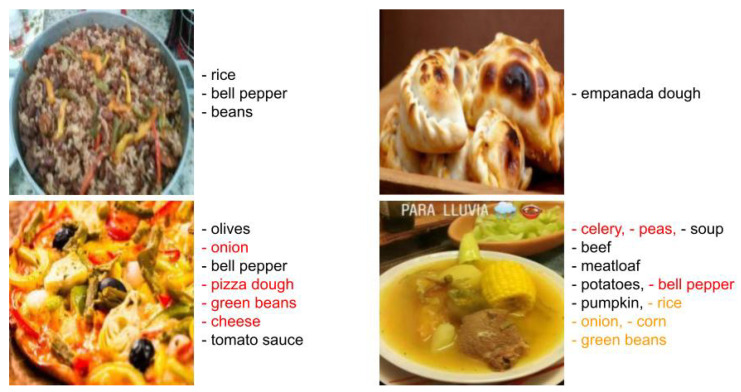
Examples of results obtained in MLChileanFood using the AFCM method. True positives, false positives, and false negatives are in black, red, and orange colors, respectively.

Some examples of successes and failures of the prediction provided by AFCM in MLChileanFood are presented in Figure 7. The images were selected according to the Jaccard index. The top two images correspond to those with the highest scores and the bottom two to those with median scores. It can be deduced that the model tends to work best when few ingredients are present in the image (e.g., less than 4). This is the case of the images in the first row, in which there are few and very distinguishable ingredients. Looking at the second row, some errors occur because the ingredients are not in the main content of the image (e.g., celery); there is too little quantity (e.g., peas); the visual appearance is very similar to another (e.g., bell pepper and green beans); or they are occluded by other ingredients (e.g., pizza dough). It is interesting to note that the proposed AFCM can recognize ingredients that correspond to the ground truth but that the annotator did not identify (e.g., the onion in the food image bottom right).

### 6.3. Analysis of the Ability to Handle Noisy Labels

In addition to the traditional evaluation of multitask learning models described above, this subsection analyzes the ability to handle noisy labels. For this purpose, the training data of the UNIMIB2016-N were divided into two parts: clean, which corresponds to data with clean labels, and Noisy, which corresponds to data in which at least one label is noisy. After generating the prediction on both subsets, the results were calculated in terms of $F_{1}$ and JI and reported in Table 5. As can be seen, AFCM offers the worst performance in both clean and noisy subsets, which is positive as it means that the model tends to prevent overfitting and avoids memorization of noisy labels.

### 6.4. Evaluation of the Robustness to Data Perturbation

**Figure 8:**
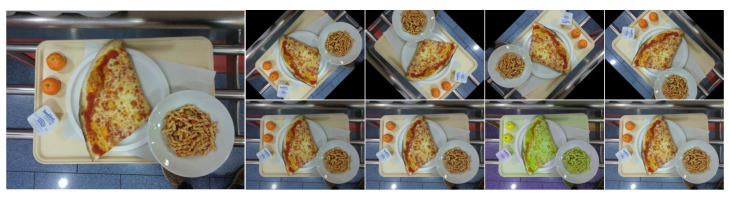
Data transformation was applied to evaluate the robustness of the model prediction. The left column is the original image; the first row represents the four rotations used (from left to right: 45, 135, 225, and 315); and the second row represents the color adjustment (from left to right: brightness, saturation, hue, and gamma).

Robustness advantages have been observed when using the posterior probability distribution in deep neural methods to provide predictions. For this reason, the proposed PAFCM, which incorporates the Laplace approximation to compute the posterior probability distribution, has been evaluated. In addition, the evaluation of AFCM using MC-Dropout is considered for comparative purposes. For the latter case, we include in the AFCM a Dropout layer with a probability of 0.1 after each residual block. This configuration was used in both cases: AFCM + Laplace and AFCM + MC-Dropout. Both are used in inference time as a post-hoc method for the proposed AFCM method. Table 6 summarizes the results in terms of the $F_{1}$ score and Jaccard index (JI), considering various data augmentation strategies for perturbing the input data (see Figure 8). As can be seen in this table, the performance between AFCM concerning PAFCM is comparable when using the Laplace approximation but decreases considerably with MC-Dropout. Unlike Laplace, the MC-Dropout prediction is made by several thin versions of the original model. This could be one reason why, for this task, the thin models achieve an under-fitting of the data distribution, and calculating the mean of all of them to obtain the final prediction does not achieve a gain in performance. Since AFCM with Laplace provides better performance, the rest of the comparison is performed based on this model.

Four rotations and four color adjustments were performed on the data to analyze the behavior of the model in response to this perturbation. In some cases, the changes are imperceptible, but they affect the performance of the model just the same. Changes in rotation and hue make a big difference in performance compared to using the original data. Both are reasonable due to the characteristics of UNIMIB2016-N and the nature of the food itself, where color is one of the most important characteristics. In most cases, PAFCM achieves a small improvement in performance concerning AFCM, which in some cases is more than 0.5%. If computational resources are not a constraint, it can be considered a variant of the proposed method to increase robustness a little.

### 6.5. Ablation Study

Table 7 shows the performance of the models in UNIMIB2016-N when using Dropout with a probability of 0.1 after each residual block during training. As is well known, Dropout acts as a regularization and thus helps to alleviate the overfitting problem. Since UNIMIB2016-N is a small dataset (it has only 650 images for training), the model is prone to memorizing the data and losing generalization ability. This phenomenon can be seen in the results, where in AFM and AFCM, a great improvement was evident with the incorporation of Dropout. With or without Dropout AFCM is much better than AFM on UNIMIB2016-N.

## 7. Discussion

This article addressed the problem of learning with multi-label noise in the context of visual food analysis. Previous studies focused mainly on single-label recognition, but a dish does not always have a specific name but is made up of several food items, which limits the scope of these methods. From the literature review, the AFM method was selected due to its good results in single-label food recognition and its ease of extension to the multi-label problem. Although most of the modules are compatible with the task at hand, in this work, the MixUp module is adapted based on the hypothesis that the soft labels resulting from MixUp should be treated differently depending on the degree of overlap of ingredients in one image concerning the area in which the food is located in the other image. In an unsupervised manner, using Grad-CAM++, the location of each ingredient is estimated after each iteration and taken into account in the proposed AFCM method to set the individual attention weights of each ingredient to finally form the soft labels.

The analysis of the results reveals that the proposed AFCM method offers significantly better performance compared to other methods, both on the UNIMIB2016-N and the MLChileanFood datasets. Specifically, in UNIMIB2016-N, AFCM notably outperforms the baseline ResNet-50 model, with improvements of over 15% in terms of $F_{1}$ and Jaccard index. Furthermore, AFCM demonstrates a good ability to handle noisy labels, being less prone to overfitting and reducing the memorization of noisy labels. This enhanced performance suggests that AFCM is more effective for learning from noisy multi-labels, which is crucial for accurately classifying ingredients in food images.

The robustness of AFCM and the probabilistic variant PAFCM was evaluated by applying data transformation techniques to the original data. It became evident that deep learning models are susceptible to small perturbations (changes in rotation or color features). PAFCM presents a better response to the disturbances evaluated, being slightly better than AFCM. However, PAFCM requires computing several forward passes to estimate the predictive posterior, which increases the computational resources required in inference time. Surprisingly, although MC dropout is frequently used for Bayesian inference, it performed poorly in the AFM method.

In summary, the results support the effectiveness of the proposed AFCM method under noisy labeling conditions. The findings presented suggest that AFCM provides encouraging performance to address practical applications related to food image analysis, such as automated dish classification, ingredient identification, and nutritional assessment. Furthermore, they underscore the importance of considering label noise during the development of machine learning models for image classification tasks. Additionally, when data are sparse, techniques such as dropout could help improve generalization.

### Limitations

In the proposed AFCM method, the adaptation of the MixUp module of AFM based on the analysis of activation maps produced remarkable results on the noisy multi-label learning problem. However, we have identified some limitations that can be addressed as possible research directions.

- **Computational Complexity:** In AFCM, extracting the activation maps after each iteration meant an increase in training time of approximately two times for UNIMIB2016-N and five times for MLChileanFood, which depends on the number of labels the images have. This does not affect the inference time but could limit the scalability if more images and labels per image are used for training. The bottleneck of AFCM occurs during the extraction process of food or ingredient regions. Therefore, designing a fast and accurate algorithm for unsupervised object localization may be a possible research direction to improve AFCM scalability.
- **Long-Tailed Food Recognition Problem:** The AFCM performance improvement was greatest for food classes that appear most frequently in the images. Particularly in food datasets, the problem of long-tailed class distribution is frequently present because of a high data imbalance, where few classes appear in most images and most classes appear in only a few images. Providing AFCM with the capacity to deal with imbalanced data in the food domain by resampling images with infrequent ingredients or managing it in the loss function could be an interesting future research direction.
- **Precision in Food Area Estimation:** One of the main components of the proposed AFCM methods is the use of Grad-CAM++ to detect the food areas. Grad-CAM++ was developed with the purpose of getting a visual explanation of the model prediction. However, they have also been successfully used as unsupervised object location [40] and segmentation [46]. Segmenting food images is a hard problem, even for supervised methods. In a semi-controlled food data acquisition system (such as UNIMIB2016), the activation maps obtained by Grad-CAM++ provide a good overview of where food is present. In an uncontrolled food data acquisition system (such as MLChileanFood), the quality of the activation maps may be less accurate. Although the food areas are probably less accurate, AFCM was able to outperform AFM in this dataset. Having a better method to estimate the food areas can lead to further improvement in the performance achieved.

## 8. Conclusions

In conclusion, this article addressed the problem of noisy multi-label learning using a novel method that is based on AFM and leverages the activation maps obtained through Grad-CAM++ to improve the generation of soft labels during the MixUp stage. The proposed AFCM was validated on two food datasets, outperforming benchmark models such as ResNet-50, AFM, HLC, and CLMSU. AFCM exhibited more than 60% in terms of the $F_{1}$ score and 40% in terms of the Jaccard index in both, UNIMIB2016-N and MLChileanFood. The ability to handle noisy labels was also demonstrated, providing poor performance on this type of data during training and achieving better performance than the rest of the methods during testing. Although the results achieved are far from perfect, we aim to continue contributing to this challenge to provide an accurate method without requiring an excessive and costly annotation process. In addition to addressing the limitation detected, in future works we will address noisy multi-label learning by designing a method based on novel deep learning architecture, such as Visual Image Transformers VIT. The motivation for exploring VIT is due to the ability of these methods to effectively capture the overall image context through self-attention mechanisms and also because they can can be efficiently used for object localization through the self-supervised transformer features. With this method, we intend to continue improving the results and thus make their application in real-life solutions possible.

## Figures and Tables

**Table 1:** Multi-label classification performance on UNIMIB2016-N dataset.

Method	R	P	$F_{1}$	JI
ResNet-50	0.2948 ± 0.0156	0.9550 ± 0.0114	0.4503 ± 0.0190	0.2907 ± 0.0157
AFM *	0.3246 ± 0.0075	0.9553 ± 0.0114	0.4846 ± 0.0095	0.3198 ± 0.0083
AFCM	0.4570 ± 0.0054	0.9358 ± 0.0053	0.6141 ± 0.0056	0.4432 ± 0.0058

* ML classification using AFM from the authors’ implementation: https://github.com/kaiwang960112/AFM, accessed on 1 October 2023.

**Table 2:** Multi-label classification performance on MLChileanFood dataset.

Method	R	P	$F_{1}$	JI
ResNet-50	0.4694 ± 0.0029	0.7332 ± 0.0047	0.5724 ± 0.0032	0.4009 ± 0.0031
AFM *	0.5184 ± 0.0064	0.7032 ± 0.0075	0.5967 ± 0.0018	0.4253 ± 0.0019
AFCM	0.5006 ± 0.0054	0.7500 ± 0.0034	0.6004 ± 0.0030	0.4290 ± 0.0031

* ML classification using AFM from the authors’ implementation (accessed on 1 October 2023): https://github.com/kaiwang960112/AFM.

**Table 3:** Multi-label classification performance on the UNIMIB2016-N and MLChileanFood dataset obtained by the AFCM method and SoA methods. The best results for each metric are in boldface.

Dataset	Method	R	P	$F_{1}$	JI
	HLC [17]	0.3618 ± 0.0181	0.8111 ± 0.0464	0.4995 ± 0.0113	0.3330 ± 0.0101
UNIMIB2016-N	CLMSU [18]	**0.5486**±**0.0004**	0.6161 ± 0.0008	0.5804 ± 0.0005	0.4089 ± 0.0005
	AFCM	0.4570 ± 0.0054	**0.9358**±**0.0053**	**0.6141**±**0.0056**	**0.4432**±**0.0058**
	HLC [17]	0.3664 ± 0.0164	0.4480 ± 0.0211	0.4025 ± 0.0024	0.2519 ± 0.0019
MLChileanFood	CLMSU [18]	**0.5958**±**0.0008**	0.5995 ± 0.0008	0.5976 ± 0.0005	0.4262 ± 0.0005
	AFCM	0.5006 ± 0.0054	**0.7500**±**0.0034**	**0.6004**±**0.0030**	**0.4290**±**0.0031**

**Table 4:** Statistical significance results when evaluating the proposed AFCM against ResNet-50, AFM, HLC, and CLMSU, according to Bonferroni-Dunn tests. For this evaluation, the $F_{1}$ was calculated at the sample level for each method on UNIMIB2016-N and MLChileanFood datasets. ** = *p*-value ≤ 0.01, * = *p*-value ≤ 0.05 and NS = Not Significant.

	ResNet-50	AFM	HLC	CLMSU
UNIMIB2016-N	**	**	**	*
MLChileanFood	**	NS	**	**

**Table 5:** Performance on UNIMIB2016-N training set for data with clean and noisy labels.

Method	Clean		Noisy	
	*F*_1_	JI	*F*_1_	JI
ResNet-50	0.8310	0.7109	0.1294	0.0692
AFM *	0.7923	0.6560	0.0657	0.0334
AFCM	0.7264	0.5704	0.0293	0.0149

* ML classification using AFM from the authors’ implementation: https://github.com/kaiwang960112/AFM, accessed on 1 October 2023.

**Table 6:** Performance for PAFCM on UNIMIB2016-N when the input data perturbed.

Method	Perturbation	$F_{1}$	JI
AFCM	-	0.6119	0.4408
PAFCM (MC-dropout)	-	0.5593	0.3882
PAFCM (Laplace)	-	0.6123	0.4418
AFCM	Rotation (45)	0.5170	0.3487
	Rotation (135)	0.4477	0.2884
	Rotation (225)	0.4557	0.2951
	Rotation (315)	0.5133	0.3453
	Brightness (0.9)	0.6067	0.4354
	Saturation (0.9)	0.6070	0.4357
	Hue (0.1)	0.3222	0.1920
	Gamma (0.9)	0.6097	0.4385
PAFCM (Laplace)	Rotation (45)	0.5216	0.3528
	Rotation (135)	0.4526	0.2986
	Rotation (225)	0.4603	0.2989
	Rotation (315)	0.5142	0.3461
	Brightness (0.9)	0.6098	0.4387
	Saturation (0.9)	0.6131	0.4420
	Hue (0.1)	0.3233	0.1920
	Gamma (0.9)	0.6099	0.4387

**Table 7:** Multi-label classification performance in UNIMIB2016-N when considering or disregarding dropout (DO).

Method	DO	R	P	$F_{1}$	JI
AFM	N	0.3668 ± 0.0175	0.9496 ± 0.0037	0.5289 ± 0.0180	0.3598 ± 0.0165
	Y	0.4180 ± 0.0087	0.9181 ± 0.0080	0.5744 ± 0.0076	0.4030 ± 0.0074
AFCM	N	0.4247 ± 0.0078	0.9550 ± 0.0044	0.5882 ± 0.0072	0.4167 ± 0.0072
	Y	0.4570 ± 0.0054	0.9358 ± 0.0053	0.6141 ± 0.0056	0.4432 ± 0.0058

## Data Availability

The original UNIMIB2016 dataset is publicly available at [47]. The UNIMIB2016 noisy label annotations (UNIMIB2016-N) and the proposed MLChileanFood dataset are available from the corresponding author upon reasonable request.
